# Astrocyte senescence promotes glutamate toxicity in cortical neurons

**DOI:** 10.1371/journal.pone.0227887

**Published:** 2020-01-16

**Authors:** Chandani Limbad, Tal Ronnen Oron, Fatouma Alimirah, Albert R. Davalos, Tara E. Tracy, Li Gan, Pierre-Yves Desprez, Judith Campisi

**Affiliations:** 1 Buck Institute for Research on Aging, Novato, California, United States of America; 2 Comparative Biochemistry Graduate Program, University of California, Berkeley, California, United States of America; 3 Gladstone Institute of Neurological Disease, San Francisco, California, United States of America; 4 Department of Neurology, Weill Institute of Neuroscience, University of California, San Francisco, California, United States of America; 5 California Pacific Medical Center, San Francisco, California, United States of America; 6 Lawrence Berkeley National Laboratory, Berkeley, California, United States of America; National Center for Geriatrics and Gerontology, JAPAN

## Abstract

Neurodegeneration is a major age-related pathology. Cognitive decline is characteristic of patients with Alzheimer’s and related dementias and cancer patients after chemo- or radio-therapies. A recently emerged driver of these and other age-related pathologies is cellular senescence, a cell fate that entails a permanent cell cycle arrest and pro-inflammatory senescence-associated secretory phenotype (SASP). Although there is a link between inflammation and neurodegenerative diseases, there are many open questions regarding how cellular senescence affects neurodegenerative pathologies. Among the various cell types in the brain, astrocytes are the most abundant. Astrocytes have proliferative capacity and are essential for neuron survival. Here, we investigated the phenotype of primary human astrocytes made senescent by X-irradiation, and identified genes encoding glutamate and potassium transporters as specifically downregulated upon senescence. This down regulation led to neuronal cell death in co-culture assays. Unbiased RNA sequencing of transcripts expressed by non-senescent and senescent astrocytes confirmed that glutamate homeostasis pathway declines upon senescence. Our results suggest a key role for cellular senescence, particularly in astrocytes, in excitotoxicity, which may lead to neurodegeneration including Alzheimer’s disease and related dementias.

## Introduction

Cellular senescence entails a permanent cell cycle arrest, and is induced in response to several types of stresses, including telomere shortening, DNA damage, oncogene activation and mitochondrial dysfunction [1]. Senescent cells are detected in culture and *in vivo* by several markers, including senescence-associated beta-galactosidase (SA-β-gal), upregulation of p16^INK4a^, and the senescence-associated secretory phenotype (SASP), which includes the secretion of High Mobility Group Box 1 (HMGB1), and downregulation of lamin B1 (LMNB1) [2]. Senescence has been studied in several cell types, including fibroblasts, epithelial cells, muscle cells, hepatocytes and endothelial cells [3–9]. Importantly, previous studies have demonstrated a key role for cellular senescence in aging and several age-related pathologies, including neurodegenerative diseases [2, 10–12]. However, relatively less is known about the potential role of senescence in the brain.

Among the essential cell types in the brain, astrocytes are the most abundant population. Astrocytes retain proliferative capacity, and their functions are crucial for neuron survival [13]. Astrocytes are critical for mediating ion homeostasis, growth factor responses and neurotransmitter functions in the brain [14]. Previous studies showed that astrocyte dysfunction is associated with multiple neurodegenerative diseases, including amyotrophic lateral sclerosis, Alzheimer’s disease (AD), Huntington’s disease (HD) and Parkinson’s disease (PD) [15, 16]. Importantly, senescent astrocytes were identified in aged and AD brain tissue [11], and other studies identified several factors that are responsible for inducing senescence in astrocytes [11, 15, 17]. These studies reported a link between an inflammatory environment and neurodegenerative diseases, but how astrocyte senescence might alter brain function in general remains unclear.

Here, we characterize the senescent phenotype of human primary astrocytes. We used X-irradiation to induce senescence in astrocytes, and real-time PCR, western blotting, ELISA and cell-based assays to characterize the senescent phenotype of these astrocytes. We determined that astrocytes undergo senescence after X-irradiation and exhibit several senescent markers, similar to those reported for fibroblasts, including upregulation of p16^INK4a^ and a SASP. Genes that regulate glutamate homeostasis as well as potassium ion and water transport are essential for normal astrocyte function. We detected a significant downregulation of several of these genes upon astrocyte senescence. Using human astrocyte and neuron co-cultures, we further established that senescent astrocytes affect the vulnerability of neurons to glutamate-induced toxicity.

## Materials and methods

### Cell culture

Human primary astrocytes and culture media were obtained from ScienCell, and cells were cultured as per the providers instructions. Purity of the astrocytes was confirmed by GFAP staining (antibody from Sigma-Aldrich, #C9205). All experiments were performed at 3% oxygen. Senescence was induced by X-ray irradiation (10 Gy).

### X-irradiation

Cells were seeded in 4-well chambers, 6-well plates or 100-mm plates, and irradiated (IR) at 320 kv and 10 mA to achieve a dose of 10 Gy. After IR, fresh media were added. Mock-irradiated cells were placed in the irradiator without power for an equivalent interval. Mock IR cells were capable of proliferation, which, if kept in culture for 14 d, become confluent and show false-positivity for some senescence markers. Irradiated cells, however, do not proliferate throughout the 14 d course of the experiment and developed senescence characteristics. To avoid false-positivity in control cells due to confluency, we processed control cells soon after mock-irradiation.

### SA-β-gal assay

SA-β-gal staining was performed using the Biovision kit (Prod# K320-250). Cells were plated at 5,000 cells/cm^2^. SA-β-gal staining was performed 24–48 h after seeding, when cells are normally in log phase growth and 60%–70% confluent, to minimize false-positive staining. Cells were washed with PBS and fixed for 5 min with 2% formaldehyde–0.2% glutaraldehyde. After another PBS wash, cells were incubated overnight at 37° C in staining solution. After washing with PBS, a minimum of 400 cells counted. Positive (blue) cells are expressed as a percentage of total cell number.

### Real time-PCR

RNA was prepared using the Isolate II RNA mini kit (Bio-52073). RNA was reverse transcribed (RT) using a Applied Biosystems (Carlsbad CA, USA) kit. Quantitative (q) RT-PCR reactions were performed using the Universal Probe Library system (Roche, South San Francisco CA, USA). For quantification, the 2^-ΔΔCp^ method was used to determine relative expression normalized to β-actin. The primer set details are in S1 Table.

### Western blot analysis

Cells were washed with PBS, scraped in 200 μl 5% sodium dodecyl sulfate, sheared through a needle and pelleted at 2,000 rpm for 5 min. Supernatants were assayed for protein concentration using the BCA protein assay kit (Pierce). Proteins were separated on 4–12% polyacrylamide gels (Biorad) and transferred to a PVDF membrane (Millipore). Membranes were blocked in 5% milk powder and incubated with primary antibody for 2 h at room temperature or overnight at 4° C. Membranes were washed with 1X tris buffered saline/tween-20 (TBS-T), incubated with HRP conjugated secondary antibodies (Invitrogen), washed with TBS-T, and detected using an Enhanced Electrochemoluminescence kit (GE Healthcare). Primary antibodies were: EAAT1 (Abcam, #ab416), Kir4.1 (Alomone Labs, #APC-035), and actin (Sigma-Aldrich, #A2228-200UL).

### Co-culture assays

Astrocytes (Sciencell, Cat#1800) were made senescent by IR as described above. After 14 days, they were treated with CMPTX-Red (Invitrogen, Cat# C34552), a fluorescent dye used to render all living astrocytes red. These cells were co-cultured with human fetal primary neurons. The red astrocytes were easily distinguished from neurons in the co-cultures. On Day 1, 40,000 human neurons (Sciencell, Cat#1520) were seeded in 24-well plates on L-Poly lysine-coated glass coverslips. On Day 3, CMPTX-Red labeled astrocytes were seeded (40,000/well) with the neurons. On Day 4, co-cultures were treated with media containing either 10 mM glutamate or control media. On Day 5, co-cultures were fixed and stained with DAPI. Fluorescence images were obtained to quantify surviving cells. CMPTX-Red+DAPI stained cells were counted as astrocytes and DAPI-only stained (blue) cells were counted as neurons.

### Enzyme-linked immunosorbent assays (ELISA) and AlphaLISA

ELISA kit to detect HMGB1 was from Neo Scientific (prod# HH0016) and used according to the manufacturer’s protocol. AlphaLISA kit to detect IL-6 was from PerkinElmer (PerkinElmer, #AL223F), used according to the manufacturer’s protocol. Conditioned media were prepared by incubating non-senescent and senescent astrocytes in serum-free medium for 24 h. Media were collected 14 d after irradiation, and ELISA results were normalized to cell number.

### Immunofluorescence

Cells were washed in PBS, fixed in 4% paraformaldehyde, permeabilized with 0.5% Triton-X 100, washed, incubated in 10% goat serum for 1 h at RT, then incubated with a GFAP-Cy3 antibody (Sigma-Aldrich, #C9205) or HMGB1 antibody (Abcam, #ab18256) at 4° C overnight. After washing, slides were incubated with Alexa conjugated secondary antibodies for 30–45 min at RT. Nuclear DNA was stained with DAPI in mounting media (Vectasheild).

### RNA-seq analysis

Human primary astrocytes from 6 different individuals were obtained from ScienCell (Cat #1800, Lot #s 17320, 17158, 16091, 16492, 16700, 18709). Cells were seeded at 5,000/cm^2^. The next day, cells were X-irradiated or mock-treated. Mock cells were cultured in serum-free media for 24 h, and RNA was isolated. X-irradiated cells were given complete media for 13 days, then cultured in serum-free media for 24 h. 14 days after X-irradiation, RNA was isolated. RNA quality and quantity was determined, then RNA samples were prepared using the Illumina Truseq kit and sent to BGI Americas Corporation for sequencing on Illumina HiSeq4000. In parallel, RNA was reverse transcribed and qRT-PCR reactions were performed for p16^INK4a^, IL-6 and IL-8.

### Statistical analyses

Error bars indicate mean ± s.e.m or SD. Significance of differences in mean values was determined using the two-tailed unpaired Student’s t test or one-way ANOVA unless otherwise mentioned. P<0.05 was considered significant. Each experiment was reproduced at least twice independently. SA-β-gal was assessed blinded.

## Results

### Irradiation induces senescence and a SASP in human primary astrocytes

**Fig 1:**
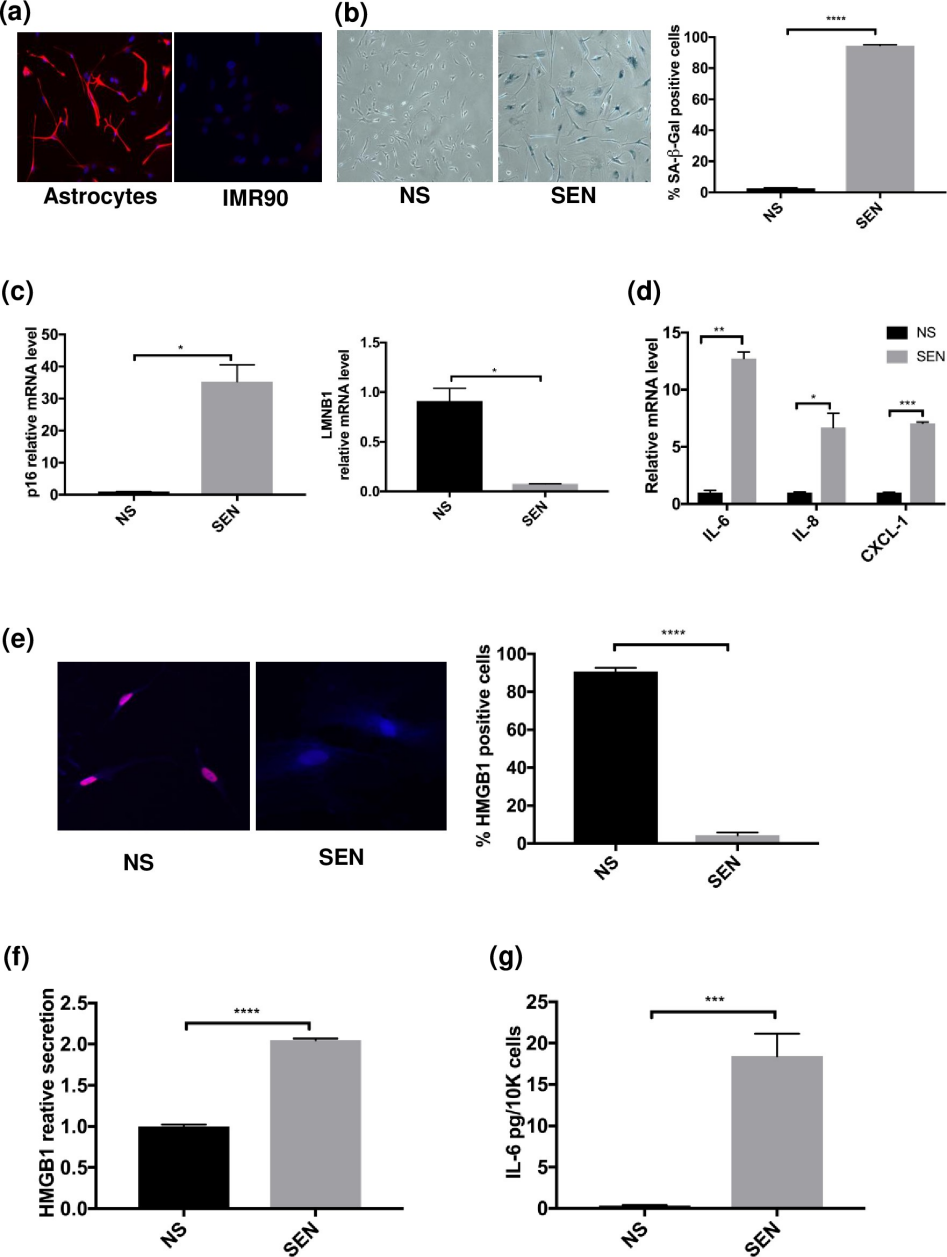
Human primary astrocytes undergo senescence and express a SASP upon X-irradiation. (a) Immunofluorescence staining for the astrocyte marker GFAP was performed on primary human astrocytes and IMR-90 fibroblasts. (b) Cellular senescence (SEN) was induced in astrocytes by X-irradiation (IR). Fourteen days after IR, SA-β-gal staining was performed on non-senescent (NS) control cells and SEN cells. Quantification is shown in the right panel. (c-d) Real-time PCR was performed on RNA samples from NS and SEN cells. (c) shows the expression of p16^INK4a^ (left) and LMNB1 (right). (d) shows the expression of three SASP factors, IL-6, IL-8 and CXCL-1. (e) High mobility group box 1 (HMGB1) immunofluorescence staining was performed on NS and SEN astrocytes. Quantification is shown in the right panel (n = 3, where n = experimental replicates). (f-g) ELISAs and AlphaLISAs were performed using conditioned media (CM) collected from NS and SEN cells; (f) shows HMGB1 ELISA results (n = 2, where n = experimental replicates), and (g) shows IL-6 AlphaLISA results (n = 3, where n = experimental replicates). For (b-d) (n = 2), shown are representative results from 3 independent experiments. For (b-g): *p<0.05, **p<0.02, ***p<0.001, ****p<0.0001 (unpaired t test).

To investigate the senescent phenotype of astrocytes, we used human primary astrocytes as described in Materials and Methods. Astrocytes purity was at least 95%, as assessed by GFAP-positivity (Fig 1A). IMR-90 fibroblasts were used as negative control for GFAP staining. Senescence was induced by ionizing X-irradiation (IR, 10 Gy) and mock irradiation was used as a control. RNA and conditioned media (CM) from non-senescent (NS) and senescence (SEN) cells were collected and fixed for staining at Day 2 and Day 14 of treatment, respectively (S1 Fig). SA-β-gal staining [18] showed that 97% of SEN astrocytes cells were positive compared to NS controls (Fig 1B). We also tested the cells several other markers of senescence. By RNA analysis, p16^INK4a^ was upregulated in SEN cells, whereas LMNB1 [19] was downregulated in SEN cells (Fig 1C). We also investigated the senescence-associated secretory phenotype (SASP), including IL-6, IL-8 and CXCL-1 [20], all of which were upregulated in SEN compared to NS cells (Fig 1D).

To determine senescence markers at the protein level, we performed immunofluorescence (IF) and ELISA for HMGB1 for NS and SEN samples. HMGB1 is a non-histone nuclear protein, and is lost from the nuclei and secreted as a damage-associated molecular pattern (DAMP) by SEN cells [21]. SEN, but not NS, astrocytes lost nuclear HMGB1 (Fig 1E). HMGB1 ELISAs on CM from NS and SEN astrocytes confirmed that HMGB1 was secreted by SEN but not NS cells (Fig 1F). AlphaLISA for IL-6 on CM from NS and SEN cells showed that IL-6 secretion was highly upregulated in SEN astrocytes versus NS cells (Fig 1G). Thus, X-irradiation-induced senescence of human astrocytes induced major senescence characteristics described for human fibroblasts [22].

### Senescence downregulates expression of genes crucial for astrocyte function

**Fig 2:**
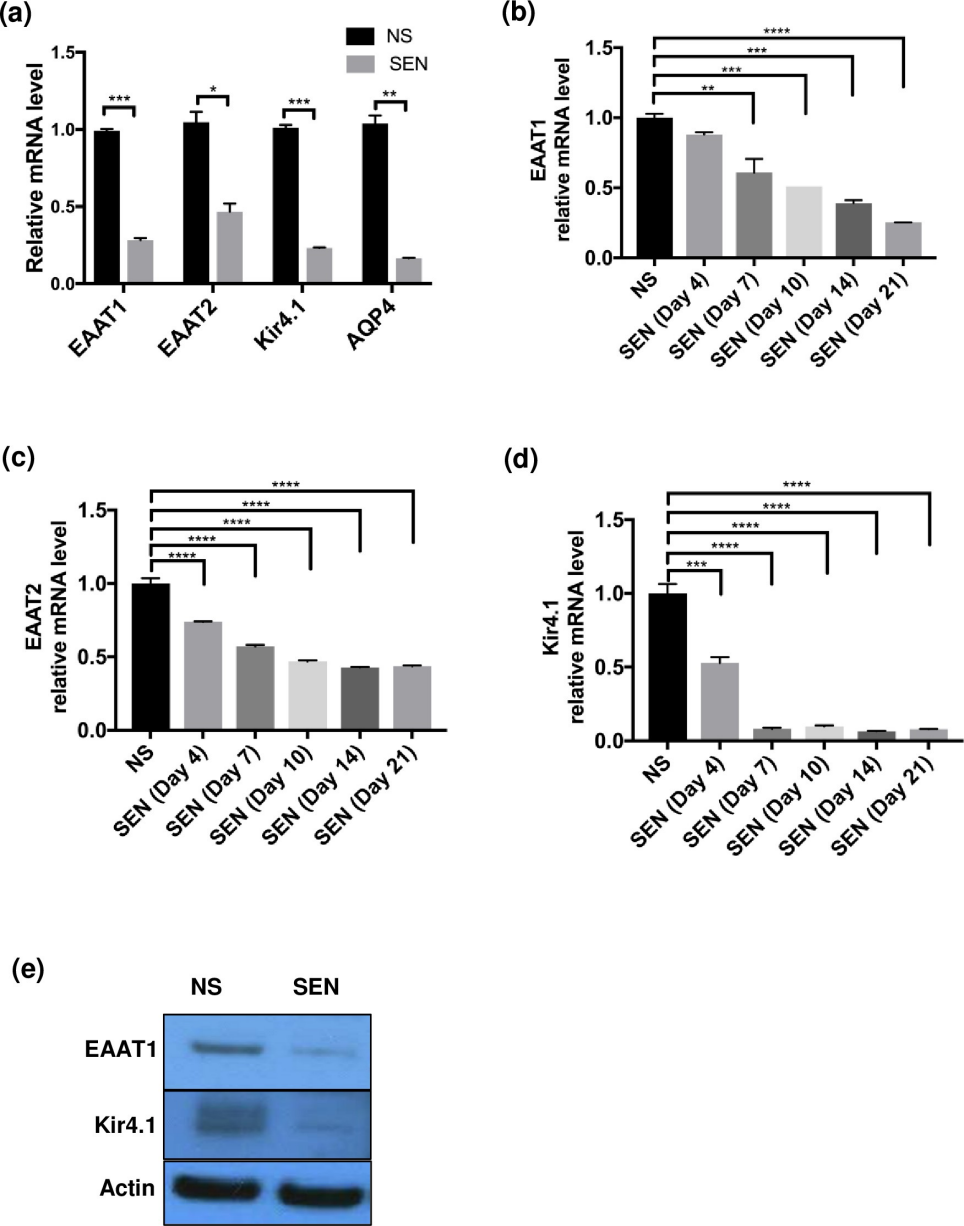
Senescence in astrocytes downregulates genes that modulate excitotoxicity. (a) Real-time PCR was performed for EAAT1, EAAT2, Kir4.1, and AQP4 mRNA using RNA samples from NS and SEN astrocytes. (b-d) Time course experiments of astrocytes after IR. Samples were collected days 7, 10, 14, 21 after treatment. Real-time PCR was performed on NS and SEN samples at the indicated times; (b) corresponds to EAAT1 gene expression, (c) corresponds to EAAT2 gene expression, and (d) corresponds to Kir4.1 gene expression. (e) Western blotting was performed using protein extracts from NS and SEN 14 days after X-irradiation. Antibodies against EAAT1 and Kir4.1 were used. For (a-e) (n = 2), shown are the representative results from 2 independent experiments. For (a): *p<0.05, **p<0.02, ***p<0.001 (unpaired t test). For (b-d): **p<0.02, ***p<0.001, ****p<0.0001 (ordinary one-way ANOVA).

Astrocytes are critical for the efficient uptake of excess glutamate from the synaptic cleft [23]. Astrocytes maintain glutamate, glutamine, potassium and water homeostasis in the brain. We therefore examined how senescence affects the expression of genes that regulate these processes in astrocytes, including Excitatory Amino Acid Transporters 1 (EAAT1) and 2 (EAAT2) (S2 Fig; [14]), potassium transporter Kir4.1 and water transporter AQP4 (also required for efficient glutamate transport [24]). By real-time PCR, the expression of all these genes were downregulated in SEN cells compared to NS astrocytes (Fig 2A).

To determine whether the downregulation of these genes was transient or persistent, we performed a time course (Day 7, 10, 14 and 21 after IR). SEN cells showed positive SA-β-gal staining compared to NS cells, and the staining in SEN cells increased with time, reaching a plateau at Day 10 (S2B Fig). RT-PCR results showed that p16^INK4a^ mRNA levels also gradually increased in SEN samples (S2C Fig). Conversely, levels of EAAT1, EAAT2 and Kir4.1 mRNA gradually declined with time, reaching their lowest levels after 14 days (Fig 2B–2D).

We also extracted proteins from NS and SEN cells 14 d after irradiation. Western analysis showed that expression of the two transporters most significantly downregulated at the mRNA level, EAAT1 and Kir4.1, also decreased at the protein level in SEN cells compared to NS cells (Fig 2E).

These results demonstrate that senescence not only induces inflammation in astrocytes, but also affects the expression of critical functional genes that modulate excitotoxicity. Using RNA sequencing (RNA-seq), we next attempted to confirm whether senescence induced by X-irradiation could indeed affect these specific pathways in astrocytes.

### RNA-seq confirms a senescence-specific deficiency in glutamate homeostasis

**Fig 3:**
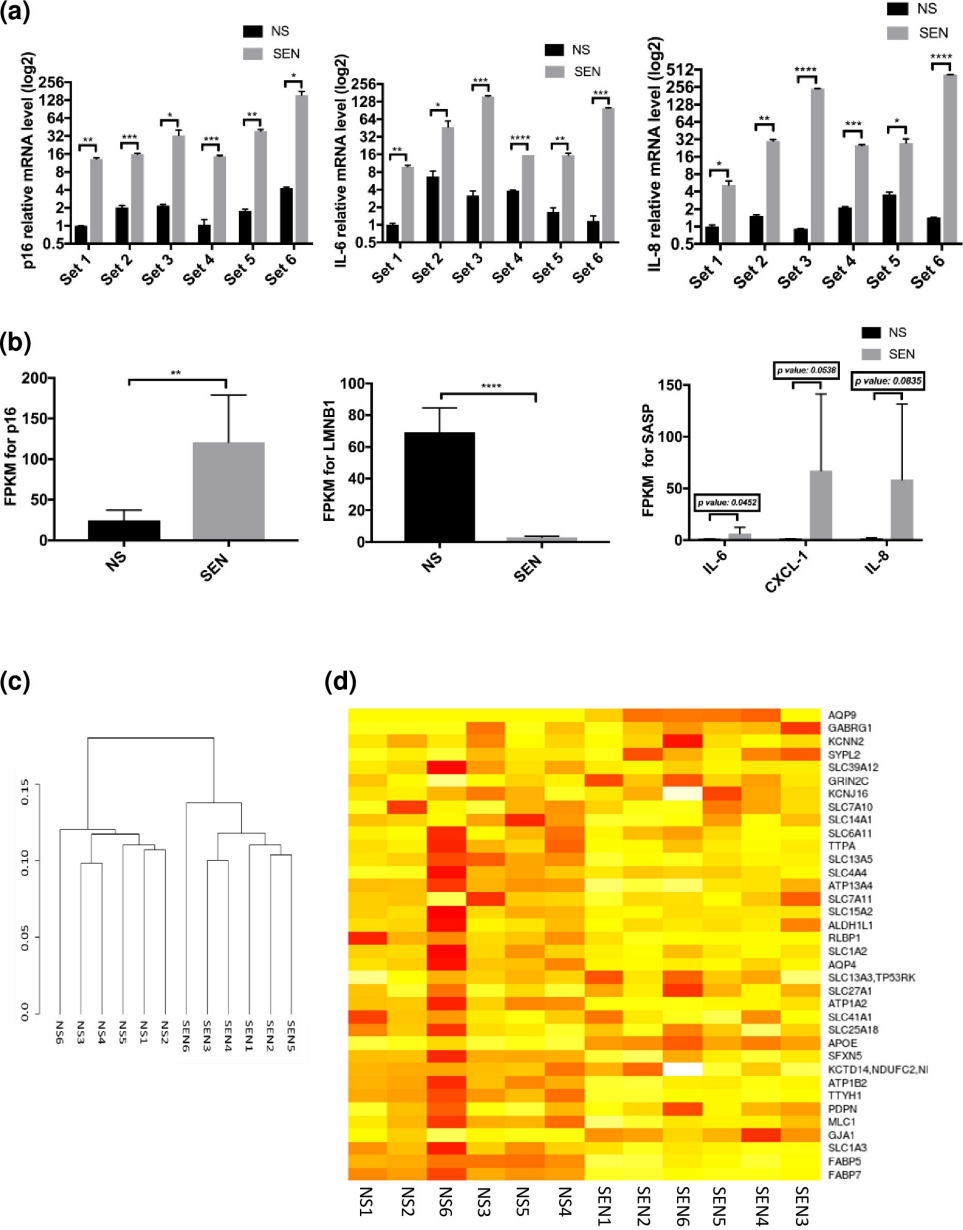
RNA-seq data reveal genes regulated in astrocytes upon senescence induction. (a) RNA extracted from SEN astrocytes corresponding to 6 different human cell strains was compared to RNA extracted from NS samples. p16^INK4a^ and SASP factors (IL-6 and IL-8) were analyzed by real-time PCR. (b) RNA-seq results (presented as Fragments Per Kilobase Million or FPKM) for the expression of various genes, i.e., p16^INK4a^ (left panel), LMNB1 (center panel) and IL-6, IL-8, CXCL-1 (right panel) were compared between NS and SEN samples. (c) Hierarchical clustering using the Jensen-Shannon distance metric was used to determine the similarity within the 6 NS samples and within the 6 SEN samples. (d) A heatmap based on FPKM of Transporter-associated Astrocyte Enriched Genes (TAEG) in all the NS and SEN samples is shown. For (a), n = 2s. For (b), n = 3. For (a, b): *p<0.05, **p<0.02, ***p<0.001, ****p<0.0001 (unpaired t test).

We used RNA-seq to compare the transcriptomes of NS and SEN astrocytes. We used astrocytes from 6 different human embryos. For SEN cells, we extracted RNA 14 days after X-irradiation. For NS cells, RNA we extracted RNA after 2 days of culture. A detailed protocol is described in Materials and Methods. Before sending the samples for sequencing, we confirmed that the senescence markers p16^INK3a^ and SASP factors IL-6 and IL-8 were elevated in SEN, compared to NS, samples (Fig 3A).

RNA-seq produced 20–25 M pair-ended, 2x150 base-long reads per sample. Trimming and filtering low quality sequences using Trimmomatics [25] resulted with 18–22 M high quality paired-end reads, which we aligned to the NCBI GRCH38 human reference genome, using TopHat2 software [26], mapping rate 89%-90% across samples (S2 Table). We used the Tuxedo protocol [27] to calculate statistical significance of differential expression.

The results showed expected changes in senescence markers. For example, p16^INK4a^ mRNA was significantly increased in SEN samples (Fig 3B, left panel), while LMNB1 mRNA was significantly decreased (Fig 3B, center panel), and mRNAs encoding SASP factors IL-6, IL-8 and CXCL-1 increased (Fig 3B, right panel). Hierarchical clustering using the Jensen-Shannon distance metric showed greater similarity among NS samples (NS1 to NS6) and among SEN samples (SEN1 to SEN6) (Fig 3C).

Using CuffDiff [27], we identified 5,809 significantly differentially expressed genes (SDE), of which 2,915 were significantly upregulated (SUR) and 2,894 were significantly downregulated (SDR) by senescence (S3 Table). We performed disease enrichment analysis on the 2,915 significantly upregulated genes and we found that these genes fell into categories such as cancer, infectious disease or AD (S4 Table). Disease enrichment analysis on the 2,894 significantly downregulated genes indicated that these genes fell into categories such as neurological disease or cancer (S5 Table).

To identify astrocyte-specific processes affected by senescence, we utilized the publicly available dataset from *NetWork Glia* (http://www.networkglia.eu/en/astrocyte), which consists of astrocyte-enriched genes from 3 published datasets (enrichment is defined as fold change ≥ log2 with a FDR ≤ 0.05). Of the 254 astrocyte-enriched genes with at least one annotated GO term, 32 genes were SUR and 91 were SDR by SEN astrocytes in our RNA-seq results (SUR-AE, SDR-AE respectively).

Using DAVID [28], SUR-AE genes with an enrichment score of 4.1, were categorized in the ‘*Extra-cellular space’* and ‘*Secreted*’ terms, and included the following 13 genes: VCAM1, MASP1, CXCL14, APOE, IL18, CTSO, CHI3L1, MFGE8, IGFBP2, FGF1, GHR, HAPLN1, SDC4. For SDR-AE genes, the first cluster, with an enrichment score of 2.4, was in the ‘*Amino-acid biosynthesis*‘ and ‘*Biosynthesis of antibiotics*’ categories, and included the following 9 genes: ACSS1, CTH, ALDOC, PHGDH, AASS, PSAT1, ACSS2, CBS, MAOB. The second cluster, with an enrichment score of 2.27, consisted of the ‘*Transporter activity’* and ‘*Transport*’ terms, and included the following 18 genes: SLC13A5, MLC1, ATP1B2, SLC15A2, FADS1, AQP4, FADS2, SPIRE1, SELENBP1, ATP1A2, DBI, SFXN5, SLC1A2, SLC1A3, TTYH1, FABP7, RLBP1, FABP5. The most enriched term for SDR-AE genes, with a FDR = 0.0015, was ‘*Negative regulation of neuron differentiation*’ with the following 7 significantly down regulated genes: HES5, SOX2, PAX6, ID4, GLI3, NR2E1, GPR37L1.

Based on the results in Fig 2, we assembled a list of 36 Transporter-associated Astrocyte Enriched Genes (TAEG) by including genes in the astrocyte-enriched dataset associated with any term containing the word ‘*Transporter*’. We then perform gene-set enrichment analysis on TAEG; this permutation analysis provided p-values of 0.08 (probability of drawing a random set of genes that more downregulated in SEN cells than the TAEG). Fig 3D shows a heatmap based on Fragments Per Kilobase of transcript per Million mapped reads (FPKM) of TAEG in NS and SEN samples. Notably, SLC1A3 corresponds to EAAT1 and SLC1A2 corresponds to EAAT2.

This analysis revealed a significant downregulation of the expression of several transporters (EAAT1, EAAT2 and Kir4.1) in all 6 SEN samples compared to NS samples, which we confirmed again by real-time PCR (S3 Fig). As an unbiased validation of our data Fig 2, these results show that senescence not only induces astrocytes to express inflammatory genes, but also reduces the expression of genes that modulate excitotoxicity.

### Senescent astrocytes confer vulnerability to glutamate toxicity on neurons

Thus, we next asked whether SEN-associated downregulation of genes encoding glutamate and potassium transporters affects glutamate uptake by astrocytes, and consequently the function of neurons. First, we applied glutamate to NS and SEN astrocytes to determine a concentration that was non-toxic to the astrocytes. At 10 mM, glutamate did not trigger any significant morphological changes in astrocytes compared to 0 mM, nor did it trigger any cell death (S4A Fig), whereas this concentration induced death of pure neuronal cultures (S4B Fig) (at 20 mM, there was substantial cell death observed in both NS and SEN astrocyte cultures). Therefore, for astrocyte/neuron co-culture experiments, we used a glutamate concentration of 10 mM.

We seeded cells for co-culture as described in Materials and Methods. After 24 h of co-culture in the absence of glutamate, neurons survived, whether cultured with NS or SEN cells. However, in the presence of 10 mM glutamate, neurons showed signs of cell death, but only when co-cultured with SEN astrocytes. No glutamate-dependent neuronal cell death was evident with neurons were cultured with glutamate and NS astrocytes. To quantify neuronal cell survival in co-culture assays, we pre-labeled the astrocytes with CMPTX-red. We initially tested the neuronal marker Beta-Tubulin III to distinguish neurons from astrocytes, but fetal astrocytes also express this marker, as reported [29].

**Fig 4:**
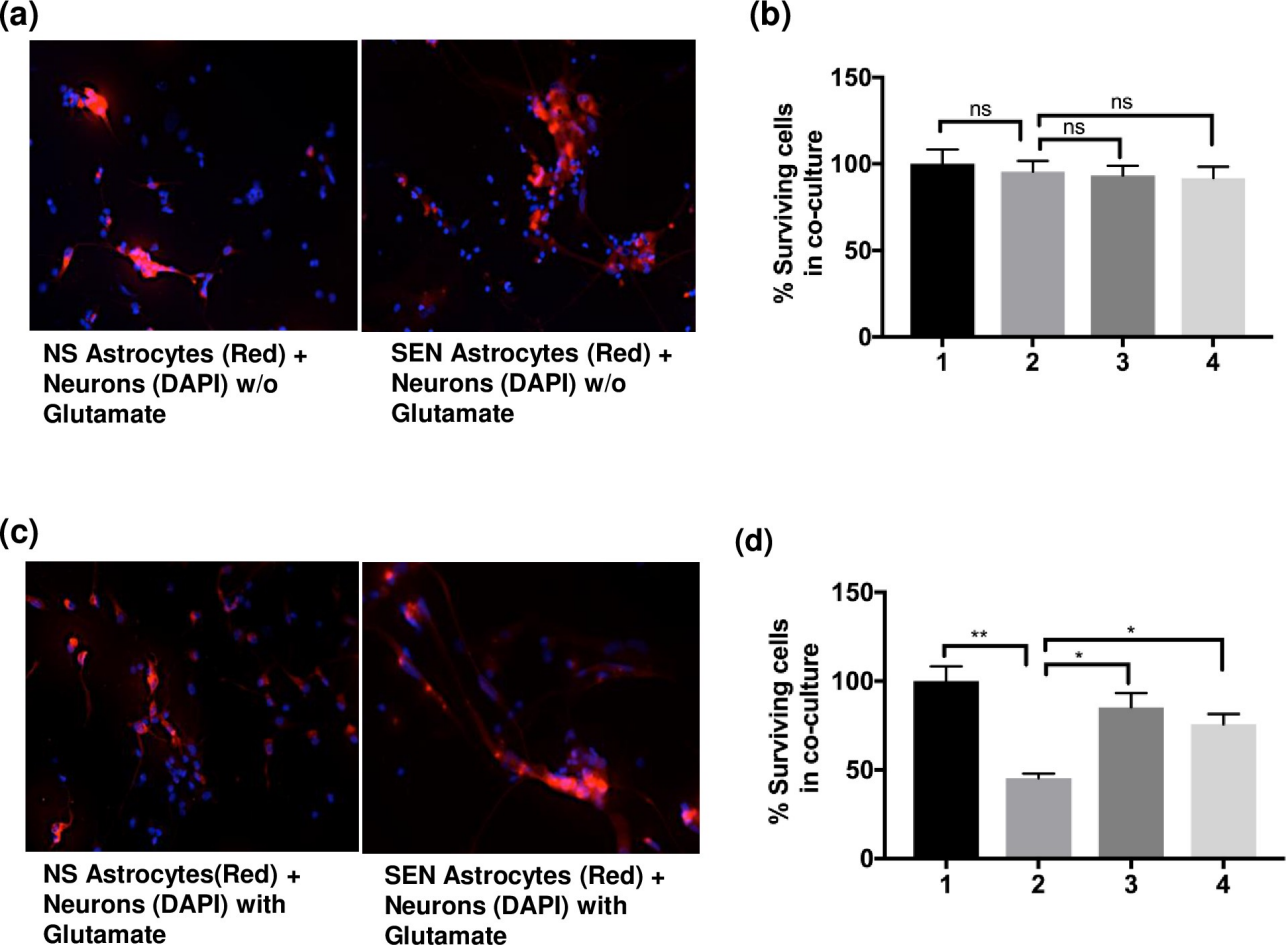
Increased neuronal death in co-cultures with SEN astrocytes dependent on glutamate. (a-b) Neurons (only DAPI-stained cells) were co-cultured with NS or SEN astrocytes (CMPTX-red + DAPI-stained cells) in neuronal media. The co-cultures were treated for 24 h with control media without glutamate. In (a) we show the fluorescent images, and in (b) the cell quantification. (c-d) Neurons were also co-cultured with NS or SEN astrocytes in neuronal media. However, here co-cultures were treated for 24 h with media containing 10 mM glutamate. In (c) we show the fluorescent images, and in (d) the cell quantification. Lane 1: surviving neurons (NS astrocytes + neurons co-cultures). Lane 2: surviving neurons (SEN astrocytes + neurons co-cultures). Lane 3: surviving astrocytes (NS astrocytes + neurons co-cultures). Lane 4: surviving astrocytes (SEN astrocytes + neurons co-cultures). For (b, d) (n = 4, where n = experimental replicates): ns = not significant, *p<0.05, **p<0.02 (unpaired t test).

We co-cultured neurons and astrocytes in the absence (Fig 4A and 4B) or presence (Fig 4C and 4D) of 10 mM glutamate. After 24 h, the cells were fixed and nuclei were stained with DAPI. Thus, red+DAPI staining identified astrocytes, whereas DAPI only staining identified neurons. In the presence of 10 mM of glutamate, almost 50% of the neurons died when co-cultured with SEN astrocytes (Fig 4D; compare Lane 2 to Lane 1). On the contrary, neurons showed no sign of cell death in the presence of glutamate when co-cultured with NS astrocytes. We conclude that SEN astrocytes do not take up glutamate efficiently, which leads to neuronal cell death.

## Discussion

Cellular senescence is a pleiotropic cell fate that prevents cancer in early life, but can also promote age-related diseases, including cancer, during aging, largely by producing pro-inflammatory SASP factors [20, 30]. Most of our understanding of the SASP derives from studies of human fibroblasts [22]. Recently, Jeon *et al*. showed that irradiation caused glioblastoma (GBM) cells, the most aggressive type of brain cancer, to undergo senescence [31, 32]. These senescent GBM cells secreted a SASP and promoted tumor cell growth.

Here, we show that human primary astrocytes also undergo senescence upon irradiation and manifest some of the classical features of senescence, including a pro-inflammatory SASP. We therefore investigated how non-cancerous senescent astrocytes might affect the brain microenvironment. We reasoned that pro-inflammatory factors secreted by astrocytes could not only promote brain cancer, but could also contribute to neurodegeneration in diseases such as AD and PD [33, 34]. Moreover, astrocyte senescence has been observed in AD patients or upon irradiation [11, 12, 34, 35]. Eliminating senescent cells could hold therapeutic promise for halting neurodegeneration. Indeed, depleting senescent astrocytes abrogates the development of neurodegenerative phenotypes associated with systemic exposure of the herbicide paraquat [36].

In addition to inflammatory factors, astrocytes play a critical role in neuronal survival [37–41]. Here, we show that senescence compromises astrocyte-specific functions, including glutamate and potassium transport. Glutamate and potassium transporters are important for maintaining the appropriate amount of glutamate in the brain [42]. Excessive glutamate causes neuronal cell death (glutamate excitotoxicity). Glutamate toxicity has been studied in various neurodegenerative diseases, including AD, PD, HD, amyotrophic lateral sclerosis (ALS) and epilepsy [16, 43, 44]. Glutamate binds to N-methyl-D-aspartate (NMDA) subtype of glutamate receptors. Almost all neurons in the CNS carry NMDA receptors, and excessive activation of these receptors leads to excitotoxicity.

In AD, amyloid β-related peptides increase glutamate release and inhibit glutamate uptake by astrocytes, leading to excitotoxicity [43, 45, 46]. Our recent preliminary results on the hippocampus of the AD mouse model J20 suggest that senescence is induced in this model, creating an inflammatory environment in the brain. We recently found a correlation between the induction of senescence and downregulation of glutamate and potassium transporters in the hippocampus, suggesting that senescence might promote glutamate toxicity in AD.

Several studies identified key genes that likely contribute to AD. These studies used AD patient samples and mouse models. In human patient samples, astrocytes are neuroprotective when glutamate transporters are highly expressed [47]. Glutamate transporter expression varies among mouse models of AD and depends on the brain region. For example, the triple transgenic (3xTg-AD) mouse shows no change in glutamate transporter expression in cortex, but decreased expression in hippocampus [48, 49]. Finally, several therapies, such as Riluzole and Ceftriaxone, designed to increase glutamate transporters improve memory function in AD humans and models [48, 50]. Our results on glutamate transporter expression and glutamate toxicity are consistent with these studies.

Interestingly, in addition to the downregulation in glutamate and potassium transport in SEN astrocytes, our RNA-seq data identified two genes, GJA1 and APOE, as upregulated in SEN compared to NS cells. GJA1 encodes the gap junction protein alpha 1 (also called connexin 43) that participates in forming channels between cells. Elevated GJA1 immunoreactivity was found at sites of amyloid plaques in AD [51]. APOE encodes apolipoprotein E, which participates in fat metabolism and plaque formation in the brain, and is important in AD since the APOE4 allele predisposes to late-onset familial AD [52, 53]. The increased expression of these two proteins in senescent astrocytes may exacerbate the pathological microenvironment of the brain in patients with AD.

Excitotoxicity has been documented in other neurodegenerative diseases [24, 54, 55]. Downregulation of glutamate transporters in astrocytes has been reported in epilepsy and ALS [44]. Moreover, decreased glutamate transporters are thought to contribute to the progressively disturbed glutamate homeostasis in the brains of patients with HD [24, 56–58]. Potassium transporters are also necessary for efficient glutamate uptake by astrocytes and preventing excitotoxicity [24]. Our data show that senescent astrocytes display a strong downregulation of the potassium transporter, Kir4.1. Kir4.1 deficiency can contribute to excitotoxicity and neuronal dysfunction, and can have detrimental effects in HD [56]. None of these disease conditions (other than AD) have been tested for the potential presence and role of senescent cells. Finally, our results show that senescent astrocytes downregulate the water transport channel AQP4. Further experiments will be needed in order to investigate the effects of AQP4 deficiency in astrocytes.

Interestingly, senescence was induced in human fetal astrocytes by transient oxidative stress [59]. Although genes involved in neuronal and glial development and differentiation were shown to be downregulated, changes in transporter expression were not reported. However, from their differential expression analysis, it was clear that the major transporters identified in our study (EAAT1, EAAT2 and Kir4.1) were also significantly downregulated in astrocytes induced to senescence by oxidative stress. These data suggest that different means of senescence induction can trigger similar effects in human astrocytes. However, downregulation of the glutamate homeostasis gene family appears to be specific to astrocytes. We also tested EAAT1, EAAT2 and Kir4.1 expression in senescent human fibroblasts, and found that these genes were either not expressed or upregulated upon senescence induction.

For the treatment of AD and ALS, there are currently a few FDA-approved drugs that inhibit NMDA receptors and are anti-excitotoxic. Among these drugs are Memantine and Riluzole [60–62]. If senescent cells, detected in neurodegenerative diseases, are found to contribute to excitotoxicity, targeted removal of these cells could open new avenues for treatment. Senolytics are a new class of drugs used to remove senescent cells, and compounds such as ABT263 have been shown to clear senescent cells efficiently and rejuvenate stem cells in aged mouse tissues [63]. This type of compounds could be used to develop novel therapeutic approaches for neurodegenerative diseases such as AD.

## Supporting information

S1 Fig The timeline for irradiation experiments and sample collections is shown. (PDF)

S2 Fig The schematic of glutamate transport and senescence time course in astrocytes. (a) The schematic of glutamate transport in astrocytes is presented. (b) Time course experiments were performed on astrocytes after X-irradiation. Samples were collected at Day 7, 10, 14, and 21 after treatment, and SA-β-gal staining was performed on NS control cells and SEN cells. Quantification is shown in the right panel. (c) Real-time PCR was performed for p16^INK4a^ expression on NS and SEN samples at the indicated times. For (b, c) (n = 2), shown are representative results from 2 independent experiments. For (b, c): *p<0.05, **p<0.02, ****p<0.0001 (ordinary one-way ANOVA).
(PDF)

S3 Fig Transporter expression in SEN and NS astrocytes from 6 different human cell strains. SEN astrocytes from 6 different human cell strains were compared for expression of glutamate and potassium transporters on Day 14 after IR. EAAT1 and EAAT2 mRNAs (left and center panels), and Kir4.1 mRNA (right panel), were analyzed by real-time PCR in NS and SEN samples from astrocytes obtained from the six different individuals. n = 2, where n = experimental replicates, and *p<0.05, **p<0.02, ***p<0.001 (unpaired t test).
(PDF)

S4 Fig Glutamate treatment on either astrocytes or neurons. (a) NS and SEN astrocytes were used to determine the optimal concentration of glutamate to be used for co-culture assays. Cells were seeded at 5,000/cm^2^ and treated with 0, 10 or 20 mM of glutamate (Glu). (b) Pure neuronal cultures, without the presence of astrocytes, were treated with 10 mM glutamate (Glu).
(PDF)

S1 Raw Images Raw western blot images for EAAT1, Kir4.1 and Actin. (PDF)

S1 Table List of primer sequences used for Real-Time PCR. (PDF)

S2 Table Read mapping to the human genome. (PDF)

S3 Table List of significantly differentially expressed genes in RNA-Seq analysis. (XLSX)

S4 Table Disease Enrichment Analysis based on significantly upregulated genes in RNA-Seq analysis. (XLSX)

S5 Table Disease Enrichment Analysis based on significantly downregulated genes in RNA-Seq analysis. (XLSX)

## References

[1] Lecot P, Alimirah F, Desprez PY, Campisi J, Wiley C. Context-dependent effects of cellular senescence in cancer development. Br J Cancer. 2016;114(11):1180–4. Epub 2016/05/04. bjc2016115 [pii] 10.1038/bjc.2016.115 27140310 PMC4891501

[2] Rodier F, Campisi J. Four faces of cellular senescence. J Cell Biol. 2011;192(4):547–56. Epub 2011/02/16. jcb.201009094 [pii] 10.1083/jcb.201009094 21321098 PMC3044123

[3] Minamino T, Miyauchi H, Yoshida T, Ishida Y, Yoshida H, Komuro I. Endothelial cell senescence in human atherosclerosis: role of telomere in endothelial dysfunction. Circulation. 2002;105(13):1541–4. Epub 2002/04/03. 10.1161/01.cir.0000013836.85741.17 . 11927518

[4] Schnabl B, Purbeck CA, Choi YH, Hagedorn CH, Brenner D. Replicative senescence of activated human hepatic stellate cells is accompanied by a pronounced inflammatory but less fibrogenic phenotype. Hepatology. 2003;37(3):653–64. Epub 2003/02/26. 10.1053/jhep.2003.50097 S027091390214208X [pii]. . 12601363

[5] Minamino T, Yoshida T, Tateno K, Miyauchi H, Zou Y, Toko H, et al Ras induces vascular smooth muscle cell senescence and inflammation in human atherosclerosis. Circulation. 2003;108(18):2264–9. Epub 2003/10/15. 10.1161/01.CIR.0000093274.82929.22 CIR.0000093274.82929.22 [pii]. . 14557365

[6] van Deursen JM. The role of senescent cells in ageing. Nature. 2014;509(7501):439–46. Epub 2014/05/23. nature13193 [pii] 10.1038/nature13193 24848057 PMC4214092

[7] Wiley CD, Velarde MC, Lecot P, Liu S, Sarnoski EA, Freund A, et al Mitochondrial Dysfunction Induces Senescence with a Distinct Secretory Phenotype. Cell Metab. 2016;23(2):303–14. Epub 2015/12/22. S1550-4131(15)00578-1 [pii] 10.1016/j.cmet.2015.11.011 26686024 PMC4749409

[8] Zhou F, Onizawa S, Nagai A, Aoshiba K. Epithelial cell senescence impairs repair process and exacerbates inflammation after airway injury. Respir Res. 2011;12:78 Epub 2011/06/15. 1465-9921-12-78 [pii] 10.1186/1465-9921-12-78 21663649 PMC3118351

[9] Krtolica A, Parrinello S, Lockett S, Desprez PY, Campisi J. Senescent fibroblasts promote epithelial cell growth and tumorigenesis: a link between cancer and aging. Proc Natl Acad Sci U S A. 2001;98(21):12072–7. Epub 2001/10/11. 10.1073/pnas.211053698 211053698 [pii]. 11593017 PMC59769

[10] Bussian TJ, Aziz A, Meyer CF, Swenson BL, van Deursen JM, Baker DJ. Clearance of senescent glial cells prevents tau-dependent pathology and cognitive decline. Nature. 2018;562(7728):578–82. Epub 2018/09/21. 10.1038/s41586-018-0543-y 30232451 PMC6206507

[11] Bhat R, Crowe EP, Bitto A, Moh M, Katsetos CD, Garcia FU, et al Astrocyte senescence as a component of Alzheimer's disease. PLoS One. 2012;7(9):e45069 Epub 2012/09/18. 10.1371/journal.pone.0045069 PONE-D-12-08309 [pii]. 22984612 PMC3440417

[12] Chinta SJ, Woods G, Rane A, Demaria M, Campisi J, Andersen JK. Cellular senescence and the aging brain. Exp Gerontol. 2015;68:3–7. Epub 2014/10/05. S0531-5565(14)00275-7 [pii] 10.1016/j.exger.2014.09.018 25281806 PMC4382436

[13] Jakel S, Dimou L. Glial Cells and Their Function in the Adult Brain: A Journey through the History of Their Ablation. Front Cell Neurosci. 2017;11:24 Epub 2017/03/01. 10.3389/fncel.2017.00024 28243193 PMC5303749

[14] Devinsky O, Vezzani A, Najjar S, De Lanerolle NC, Rogawski MA. Glia and epilepsy: excitability and inflammation. Trends Neurosci. 2013;36(3):174–84. Epub 2013/01/10. S0166-2236(12)00205-6 [pii] 10.1016/j.tins.2012.11.008 . 23298414

[15] Bitto A, Sell C, Crowe E, Lorenzini A, Malaguti M, Hrelia S, et al Stress-induced senescence in human and rodent astrocytes. Exp Cell Res. 2010;316(17):2961–8. Epub 2010/07/14. S0014-4827(10)00342-3 [pii] 10.1016/j.yexcr.2010.06.021 . 20620137

[16] Maragakis NJ, Rothstein JD. Mechanisms of Disease: astrocytes in neurodegenerative disease. Nat Clin Pract Neurol. 2006;2(12):679–89. Epub 2006/11/23. ncpneuro0355 [pii] 10.1038/ncpneuro0355 . 17117171

[17] Mombach JC, Vendrusculo B, Bugs CA. A Model for p38MAPK-Induced Astrocyte Senescence. PLoS One. 2015;10(5):e0125217 Epub 2015/05/09. 10.1371/journal.pone.0125217 PONE-D-14-53952 [pii]. 25954815 PMC4425668

[18] Dimri GP, Lee X, Basile G, Acosta M, Scott G, Roskelley C, et al A novel biomarker identifies senescent human cells in culture and in aging skin in vivo. Proc Natl Acad Sci USA. 1995;92:9363–7. 10.1073/pnas.92.20.9363 7568133 PMC40985

[19] Freund A, Laberge RM, Demaria M, Campisi J. Lamin B1 loss is a senescence-associated biomarker. Mol Biol Cell. 2012;23(11):2066–75. Epub 2012/04/13. mbc.E11-10-0884 [pii] 10.1091/mbc.E11-10-0884 22496421 PMC3364172

[20] Coppe JP, Patil CK, Rodier F, Sun Y, Munoz DP, Goldstein J, et al Senescence-associated secretory phenotypes reveal cell-nonautonomous functions of oncogenic RAS and the p53 tumor suppressor. PLoS Biol. 2008;6(12):2853–68. Epub 2008/12/05. 08-PLBI-RA-2566 [pii] 10.1371/journal.pbio.0060301 19053174 PMC2592359

[21] Davalos AR, Kawahara M, Malhotra GK, Schaum N, Huang J, Ved U, et al p53-dependent release of Alarmin HMGB1 is a central mediator of senescent phenotypes. J Cell Biol. 2013;201(4):613–29. Epub 2013/05/08. jcb.201206006 [pii] 10.1083/jcb.201206006 23649808 PMC3653366

[22] Coppe JP, Desprez PY, Krtolica A, Campisi J. The senescence-associated secretory phenotype: the dark side of tumor suppression. Annu Rev Pathol. 2010;5:99–118. Epub 2010/01/19. 10.1146/annurev-pathol-121808-102144 20078217 PMC4166495

[23] Kimelberg HK, Nedergaard M. Functions of astrocytes and their potential as therapeutic targets. Neurotherapeutics. 2010;7(4):338–53. Epub 2010/10/01. S1933-7213(10)00111-X [pii] 10.1016/j.nurt.2010.07.006 20880499 PMC2982258

[24] Kucheryavykh YV, Kucheryavykh LY, Nichols CG, Maldonado HM, Baksi K, Reichenbach A, et al Downregulation of Kir4.1 inward rectifying potassium channel subunits by RNAi impairs potassium transfer and glutamate uptake by cultured cortical astrocytes. Glia. 2007;55(3):274–81. Epub 2006/11/09. 10.1002/glia.20455 . 17091490

[25] Bolger AM, Lohse M, Usadel B. Trimmomatic: a flexible trimmer for Illumina sequence data. Bioinformatics. 2014;30(15):2114–20. Epub 2014/04/04. btu170 [pii] 10.1093/bioinformatics/btu170 24695404 PMC4103590

[26] Kim D, Pertea G, Trapnell C, Pimentel H, Kelley R, Salzberg SL. TopHat2: accurate alignment of transcriptomes in the presence of insertions, deletions and gene fusions. Genome Biol. 2013;14(4):R36 Epub 2013/04/27. gb-2013-14-4-r36 [pii] 10.1186/gb-2013-14-4-r36 23618408 PMC4053844

[27] Trapnell C, Roberts A, Goff L, Pertea G, Kim D, Kelley DR, et al Differential gene and transcript expression analysis of RNA-seq experiments with TopHat and Cufflinks. Nat Protoc. 2012;7(3):562–78. Epub 2012/03/03. nprot.2012.016 [pii] 10.1038/nprot.2012.016 22383036 PMC3334321

[28] Huang da W, Sherman BT, Lempicki RA. Systematic and integrative analysis of large gene lists using DAVID bioinformatics resources. Nat Protoc. 2009;4(1):44–57. Epub 2009/01/10. nprot.2008.211 [pii] 10.1038/nprot.2008.211 . 19131956

[29] Draberova E, Del Valle L, Gordon J, Markova V, Smejkalova B, Bertrand L, et al Class III beta-tubulin is constitutively coexpressed with glial fibrillary acidic protein and nestin in midgestational human fetal astrocytes: implications for phenotypic identity. J Neuropathol Exp Neurol. 2008;67(4):341–54. Epub 2008/04/02. 10.1097/NEN.0b013e31816a686d . 18379434

[30] Campisi J, d'Adda di Fagagna F. Cellular senescence: when bad things happen to good cells. Nature Rev Molec Cell Biol. 2007;8:729–40. 17667954 10.1038/nrm2233

[31] Jeon HY, Kim JK, Ham SW, Oh SY, Kim J, Park JB, et al Irradiation induces glioblastoma cell senescence and senescence-associated secretory phenotype. Tumour Biol. 2016;37(5):5857–67. Epub 2015/11/21. 10.1007/s13277-015-4439-2 [pii]. . 26586398

[32] Riemenschneider MJ, Reifenberger G. Astrocytic tumors. Recent Results Cancer Res. 2009;171:3–24. Epub 2009/03/27. 10.1007/978-3-540-31206-2_1 . 19322535

[33] Morales I, Guzman-Martinez L, Cerda-Troncoso C, Farias GA, Maccioni RB. Neuroinflammation in the pathogenesis of Alzheimer's disease. A rational framework for the search of novel therapeutic approaches. Front Cell Neurosci. 2014;8:112 Epub 2014/05/06. 10.3389/fncel.2014.00112 24795567 PMC4001039

[34] Tan FC, Hutchison ER, Eitan E, Mattson MP. Are there roles for brain cell senescence in aging and neurodegenerative disorders? Biogerontology. 2014;15(6):643–60. Epub 2014/10/12. 10.1007/s10522-014-9532-1 25305051 PMC4264619

[35] Zou Y, Zhang N, Ellerby LM, Davalos AR, Zeng X, Campisi J, et al Responses of human embryonic stem cells and their differentiated progeny to ionizing radiation. Biochem Biophys Res Commun. 2012;426(1):100–5. Epub 2012/08/25. S0006-291X(12)01553-7 [pii] 10.1016/j.bbrc.2012.08.043 22917535 PMC3498829

[36] Chinta SJ, Woods G, Demaria M, Rane A, Zou Y, McQuade A, et al Cellular Senescence Is Induced by the Environmental Neurotoxin Paraquat and Contributes to Neuropathology Linked to Parkinson's Disease. Cell Rep. 2018;22(4):930–40. Epub 2018/02/02. S2211-1247(17)31929-0 [pii] 10.1016/j.celrep.2017.12.092 29386135 PMC5806534

[37] McGeer EG, McGeer PL. The importance of inflammatory mechanisms in Alzheimer disease. Exp Gerontol. 1998;33(5):371–8. Epub 1998/10/08. S0531-5565(98)00013-8 [pii]. 10.1016/s0531-5565(98)00013-8 . 9762518

[38] Ransom B, Behar T, Nedergaard M. New roles for astrocytes (stars at last). Trends Neurosci. 2003;26(10):520–2. Epub 2003/10/03. S0166-2236(03)00259-5 [pii] 10.1016/j.tins.2003.08.006 . 14522143

[39] Salminen A, Ojala J, Kaarniranta K, Haapasalo A, Hiltunen M, Soininen H. Astrocytes in the aging brain express characteristics of senescence-associated secretory phenotype. Eur J Neurosci. 2011;34(1):3–11. Epub 2011/06/09. 10.1111/j.1460-9568.2011.07738.x . 21649759

[40] Sofroniew MV, Vinters HV. Astrocytes: biology and pathology. Acta Neuropathol. 2010;119(1):7–35. Epub 2009/12/17. 10.1007/s00401-009-0619-8 20012068 PMC2799634

[41] Volterra A, Meldolesi J. Astrocytes, from brain glue to communication elements: the revolution continues. Nat Rev Neurosci. 2005;6(8):626–40. Epub 2005/07/19. 10.1038/nrn1722 [pii] 10.1038/nrn1722. . 16025096

[42] Anderson CM, Swanson RA. Astrocyte glutamate transport: review of properties, regulation, and physiological functions. Glia. 2000;32(1):1–14. Epub 2000/09/07. 10.1002/1098-1136(200010)32:1<1::AID-GLIA10>3.0CO;2-W [pii]. . 10975906

[43] Caudle WM, Zhang J. Glutamate, excitotoxicity, and programmed cell death in Parkinson disease. Exp Neurol. 2009;220(2):230–3. Epub 2009/10/10. S0014-4886(09)00414-2 [pii] 10.1016/j.expneurol.2009.09.027 . 19815009

[44] Seifert G, Schilling K, Steinhauser C. Astrocyte dysfunction in neurological disorders: a molecular perspective. Nat Rev Neurosci. 2006;7(3):194–206. Epub 2006/02/24. nrn1870 [pii] 10.1038/nrn1870 . 16495941

[45] Revett TJ, Baker GB, Jhamandas J, Kar S. Glutamate system, amyloid ss peptides and tau protein: functional interrelationships and relevance to Alzheimer disease pathology. J Psychiatry Neurosci. 2013;38(1):6–23. Epub 2012/08/17. [pii] 10.1503/jpn.110190 22894822 PMC3529221

[46] Hynd MR, Scott HL, Dodd PR. Glutamate-mediated excitotoxicity and neurodegeneration in Alzheimer's disease. Neurochem Int. 2004;45(5):583–95. Epub 2004/07/06. 10.1016/j.neuint.2004.03.007 S0197018604000555 [pii]. . 15234100

[47] Kobayashi E, Nakano M, Kubota K, Himuro N, Mizoguchi S, Chikenji T, et al Activated forms of astrocytes with higher GLT-1 expression are associated with cognitive normal subjects with Alzheimer pathology in human brain. Sci Rep. 2018;8(1):1712 Epub 2018/01/28. 10.1038/s41598-018-19442-7 29374250 PMC5786045

[48] Zumkehr J, Rodriguez-Ortiz CJ, Cheng D, Kieu Z, Wai T, Hawkins C, et al Ceftriaxone ameliorates tau pathology and cognitive decline via restoration of glial glutamate transporter in a mouse model of Alzheimer's disease. Neurobiol Aging. 2015;36(7):2260–71. Epub 2015/05/13. 10.1016/j.neurobiolaging.2015.04.005 . 25964214

[49] Kulijewicz-Nawrot M, Sykova E, Chvatal A, Verkhratsky A, Rodriguez JJ. Astrocytes and glutamate homoeostasis in Alzheimer's disease: a decrease in glutamine synthetase, but not in glutamate transporter-1, in the prefrontal cortex. ASN Neuro. 2013;5(4):273–82. Epub 2013/09/26. 10.1042/AN20130017 24059854 PMC3791522

[50] Pereira AC, Gray JD, Kogan JF, Davidson RL, Rubin TG, Okamoto M, et al Age and Alzheimer's disease gene expression profiles reversed by the glutamate modulator riluzole. Mol Psychiatry. 2017;22(2):296–305. Epub 2016/03/30. 10.1038/mp.2016.33 27021815 PMC5042881

[51] Nagy JI, Li W, Hertzberg EL, Marotta CA. Elevated connexin43 immunoreactivity at sites of amyloid plaques in Alzheimer's disease. Brain Res. 1996;717(1–2):173–8. Epub 1996/04/22. 0006-8993(95)01526-4 [pii]. 10.1016/0006-8993(95)01526-4 . 8738268

[52] Strittmatter WJ, Saunders AM, Schmechel D, Pericak-Vance M, Enghild J, Salvesen GS, et al Apolipoprotein E: high-avidity binding to beta-amyloid and increased frequency of type 4 allele in late-onset familial Alzheimer disease. Proc Natl Acad Sci U S A. 1993;90(5):1977–81. Epub 1993/03/01. 10.1073/pnas.90.5.1977 8446617 PMC46003

[53] Carter DB. The interaction of amyloid-beta with ApoE. Subcell Biochem. 2005;38:255–72. Epub 2005/02/16. 10.1007/0-387-23226-5_13 . 15709483

[54] Van Den Bosch L, Van Damme P, Bogaert E, Robberecht W. The role of excitotoxicity in the pathogenesis of amyotrophic lateral sclerosis. Biochim Biophys Acta. 2006;1762(11–12):1068–82. Epub 2006/06/30. S0925-4439(06)00082-2 [pii] 10.1016/j.bbadis.2006.05.002 . 16806844

[55] Doble A. The role of excitotoxicity in neurodegenerative disease: implications for therapy. Pharmacol Ther. 1999;81(3):163–221. Epub 1999/05/20. S0163-7258(98)00042-4 [pii]. 10.1016/s0163-7258(98)00042-4 . 10334661

[56] Tong X, Ao Y, Faas GC, Nwaobi SE, Xu J, Haustein MD, et al Astrocyte Kir4.1 ion channel deficits contribute to neuronal dysfunction in Huntington's disease model mice. Nat Neurosci. 2014;17(5):694–703. Epub 2014/04/02. nn.3691 [pii] 10.1038/nn.3691 24686787 PMC4064471

[57] Behrens PF, Franz P, Woodman B, Lindenberg KS, Landwehrmeyer GB. Impaired glutamate transport and glutamate-glutamine cycling: downstream effects of the Huntington mutation. Brain. 2002;125(Pt 8):1908–22. Epub 2002/07/24. 10.1093/brain/awf180 . 12135980

[58] Estrada Sanchez AM, Mejia-Toiber J, Massieu L. Excitotoxic neuronal death and the pathogenesis of Huntington's disease. Arch Med Res. 2008;39(3):265–76. Epub 2008/02/19. S0188-4409(07)00412-2 [pii] 10.1016/j.arcmed.2007.11.011 . 18279698

[59] Crowe EP, Tuzer F, Gregory BD, Donahue G, Gosai SJ, Cohen J, et al Changes in the Transcriptome of Human Astrocytes Accompanying Oxidative Stress-Induced Senescence. Front Aging Neurosci. 2016;8:208 Epub 2016/09/16. 10.3389/fnagi.2016.00208 27630559 PMC5005348

[60] Howard R, McShane R, Lindesay J, Ritchie C, Baldwin A, Barber R, et al Nursing home placement in the Donepezil and Memantine in Moderate to Severe Alzheimer's Disease (DOMINO-AD) trial: secondary and post-hoc analyses. Lancet Neurol. 2015;14(12):1171–81. Epub 2015/10/31. S1474-4422(15)00258-6 [pii] 10.1016/S1474-4422(15)00258-6 . 26515660

[61] Zoccolella S, Beghi E, Palagano G, Fraddosio A, Guerra V, Samarelli V, et al Riluzole and amyotrophic lateral sclerosis survival: a population-based study in southern Italy. Eur J Neurol. 2007;14(3):262–8. Epub 2007/03/16. ENE1575 [pii] 10.1111/j.1468-1331.2006.01575.x . 17355545

[62] Lacomblez L, Bensimon G, Leigh PN, Guillet P, Meininger V. Dose-ranging study of riluzole in amyotrophic lateral sclerosis. Amyotrophic Lateral Sclerosis/Riluzole Study Group II. Lancet. 1996;347(9013):1425–31. Epub 1996/05/25. S0140-6736(96)91680-3 [pii]. 10.1016/s0140-6736(96)91680-3 . 8676624

[63] Chang J, Wang Y, Shao L, Laberge RM, Demaria M, Campisi J, et al Clearance of senescent cells by ABT263 rejuvenates aged hematopoietic stem cells in mice. Nat Med. 2016;22(1):78–83. Epub 2015/12/15. nm.4010 [pii] 10.1038/nm.4010 26657143 PMC4762215

