# Supplementary figures and images for: Astrocyte senescence promotes glutamate toxicity in cortical neurons
Source: PLoS One. 2020 Jan 16;15(1):e0227887. doi: 10.1371/journal.pone.0227887 (PMC6964973; doi:10.1371/journal.pone.0227887)

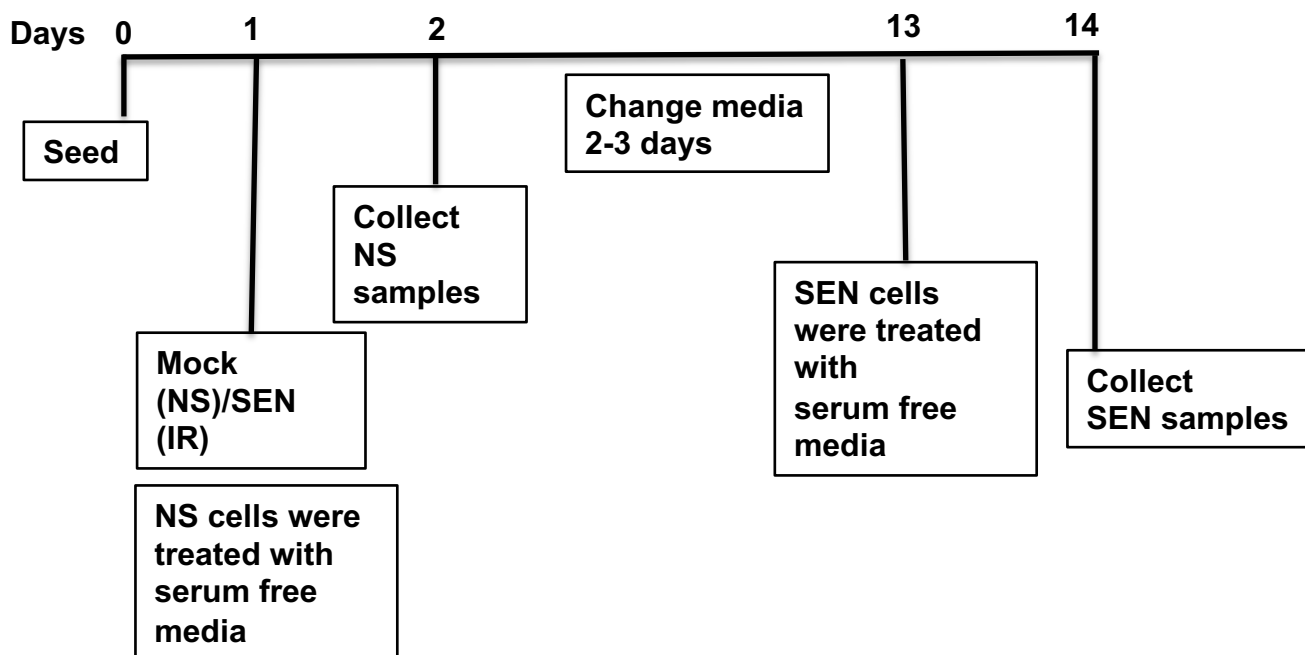

Supplement: S1 Fig — (PDF) [file pone.0227887.s001.pdf]

(a)

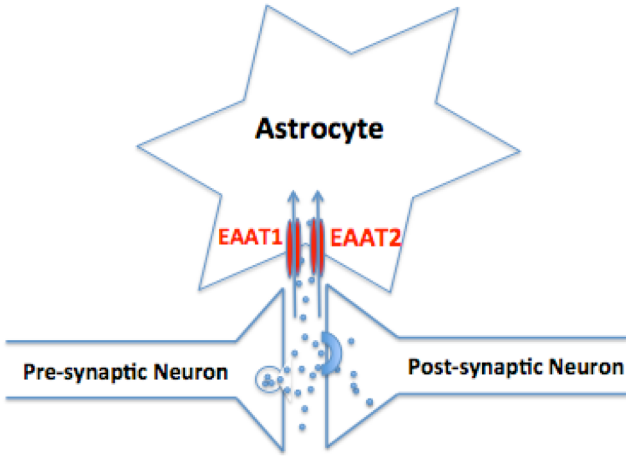

(b)

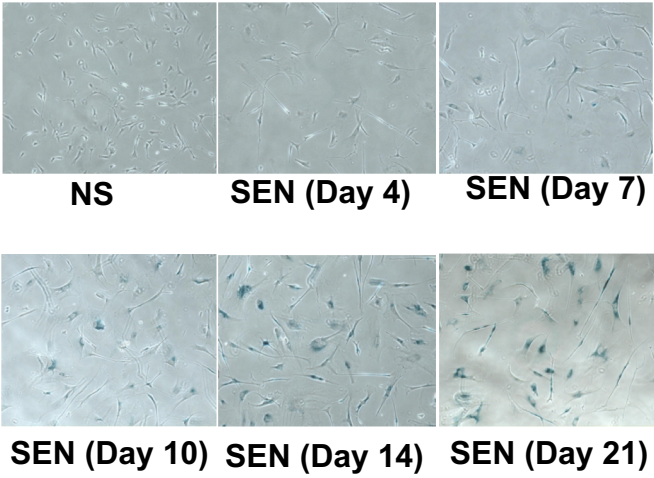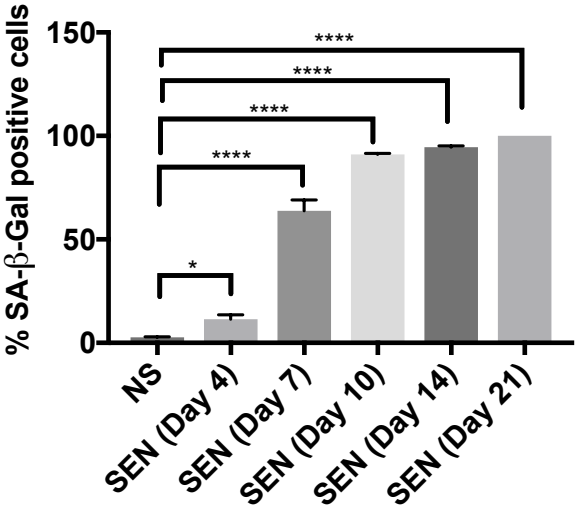

(c)

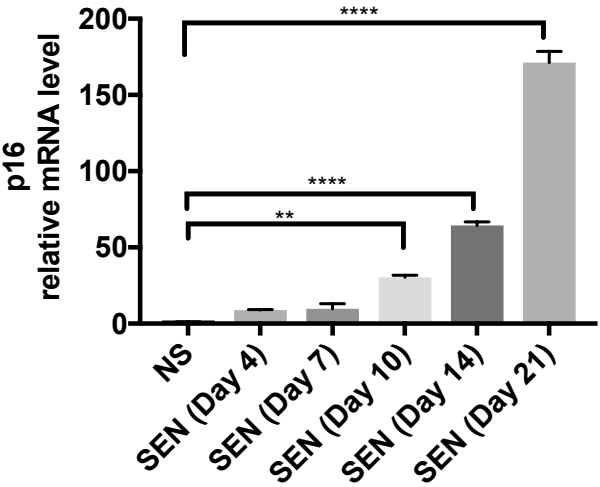

Supplement: S2 Fig — (a) The schematic of glutamate transport in astrocytes is presented. (b) Time course experiments were performed on astrocytes after X-irradiation. Samples were collected at Day 7, 10, 14, and 21 after treatment, and SA-β-gal staining was performed on NS control cells and SEN cells. Quantification is shown in the right panel. (c) Real-time PCR was performed for p16INK4a expression on NS and SEN samples at the indicated times. For (b, c) (n = 2), shown are representative results from 2 independent experiments. For (b, c): *p<0.05, **p<0.02, ****p<0.0001 (ordinary one-way ANOVA). (PDF) [file pone.0227887.s002.pdf]

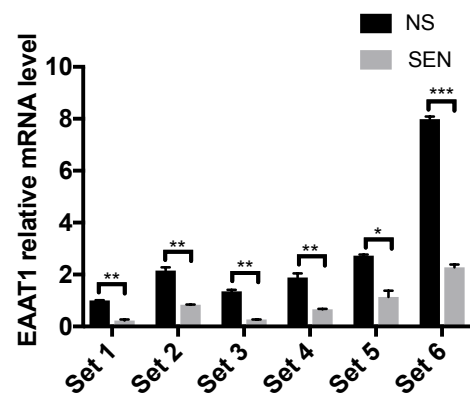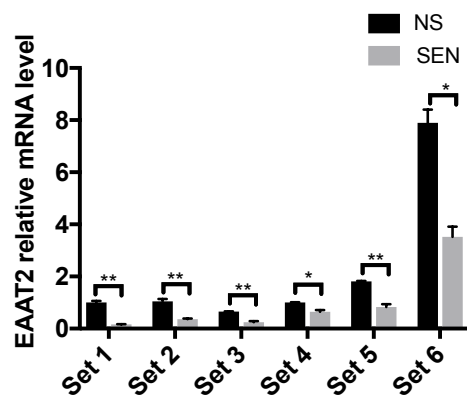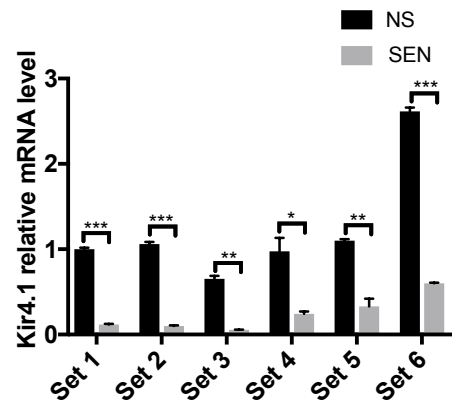

Supplement: S3 Fig — SEN astrocytes from 6 different human cell strains were compared for expression of glutamate and potassium transporters on Day 14 after IR. EAAT1 and EAAT2 mRNAs (left and center panels), and Kir4.1 mRNA (right panel), were analyzed by real-time PCR in NS and SEN samples from astrocytes obtained from the six different individuals. n = 2, where n = experimental replicates, and *p<0.05, **p<0.02, ***p<0.001 (unpaired t test). (PDF) [file pone.0227887.s003.pdf]

**(a)**

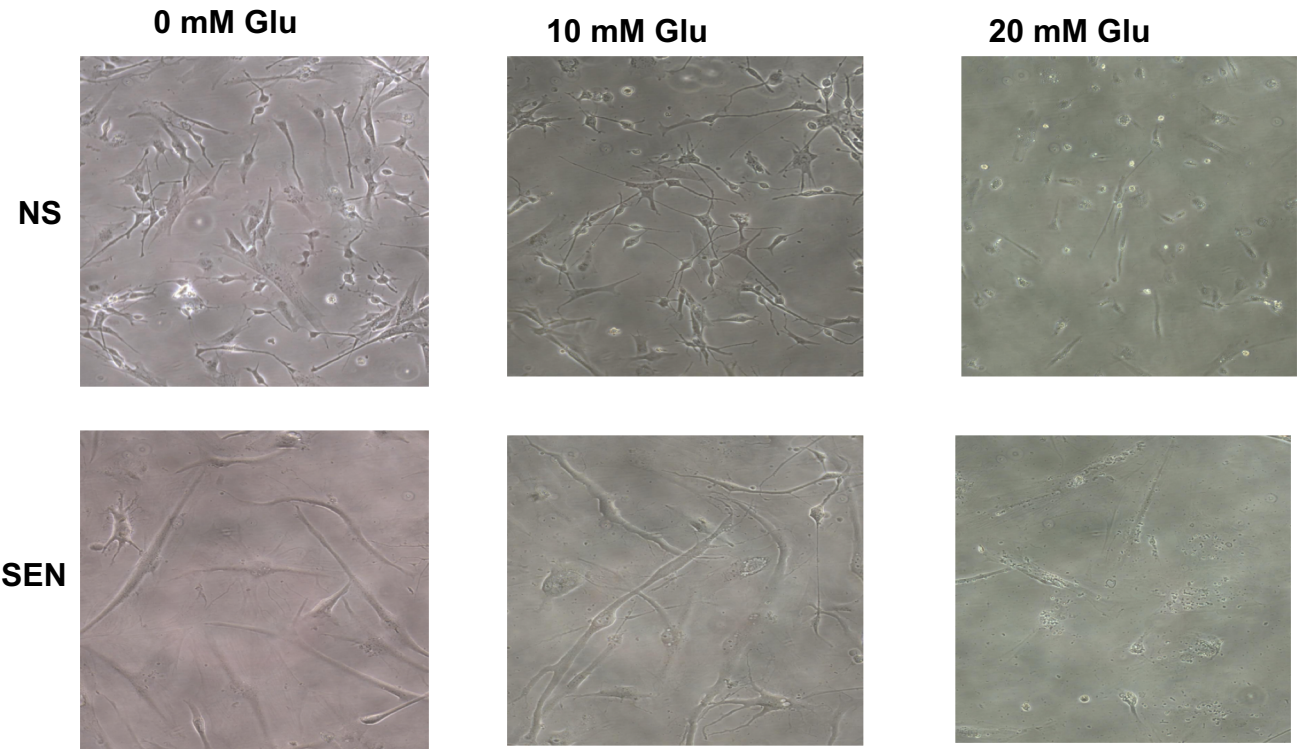

**(b)**

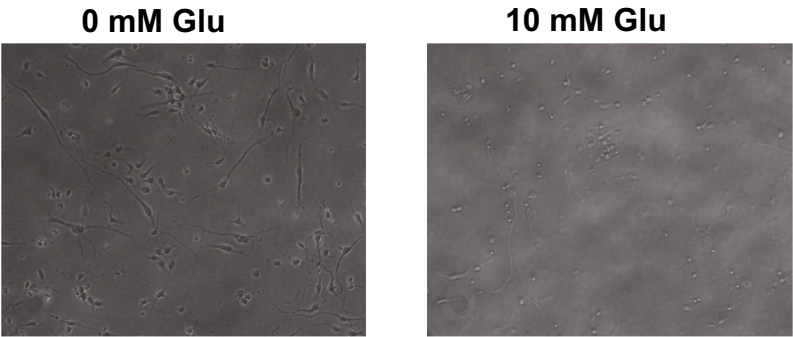

Supplement: S4 Fig — (a) NS and SEN astrocytes were used to determine the optimal concentration of glutamate to be used for co-culture assays. Cells were seeded at 5,000/cm2 and treated with 0, 10 or 20 mM of glutamate (Glu). (b) Pure neuronal cultures, without the presence of astrocytes, were treated with 10 mM glutamate (Glu). (PDF) [file pone.0227887.s004.pdf]

EAAT1

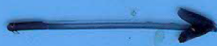

hASWo MS(NS)  
(SEN) ZR-14  
X

-75

-50

-35

-25

kin 4.1

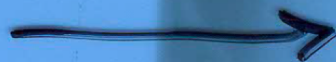

HAst20 (NS)  
(SEN) IR-14

X

-50

-37

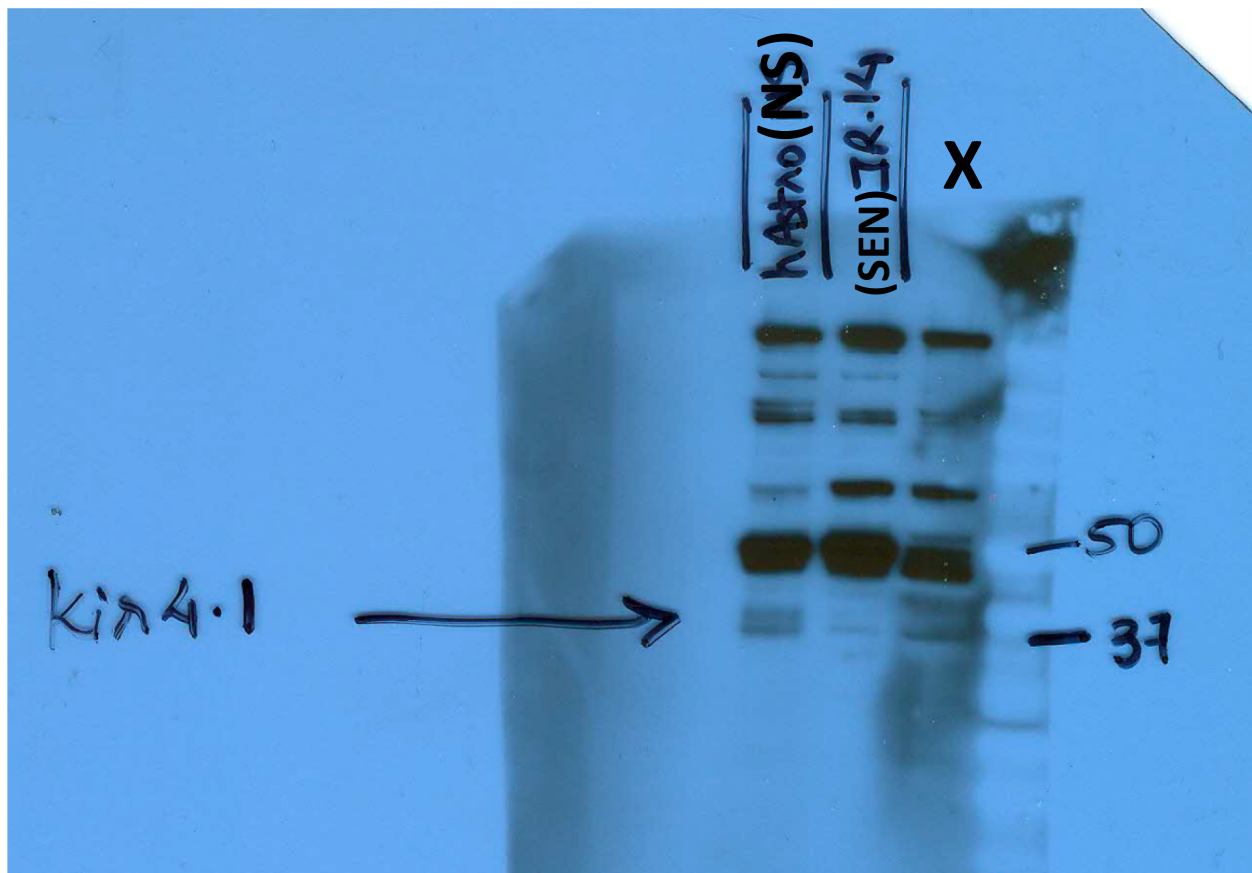

Actin →

hAstro NS(NS)  
(SEN) IR-14  
**X**

— 75  
— 50  
— 37  
— 25

Supplement: S1 Raw Images — (PDF) [file pone.0227887.s005.pdf]
