# Supplementary material for: Astrocyte senescence promotes glutamate toxicity in cortical neurons
Source: PLoS One. 2020 Jan 16;15(1):e0227887. doi: 10.1371/journal.pone.0227887 (PMC6964973; doi:10.1371/journal.pone.0227887)
Supplement: S1 Table — (PDF) [file pone.0227887.s006.pdf]

	Human Primers	UPL Probe	Forward Primer	Reverse Primer
1	Actin-1	64	Ccaaccgcgagaagatga	Tccatcacgatgccagtg
2	p16	67	Gagcagcatggagccttc	Cgtaactattcggtgcgttg
3	LMNB1	3	Gtgctgcgagcaggagac	Ccattaagatcagattccttcttagc
4	IL-6	45	Gcccagctatgaactccttct	Gaaggcagcaggcaacac
5	IL-8	72	Agacagcagagcacacaagc	Atggttccttccggtggt
6	IL-1b	78	Tacctgtcctgcgtgttgaa	Tctttgggtaatttttgggatct
7	TGF-A	64	Cattgtccatgcctcaggata	Gatttctagaagaaaaatccccaaa
8	CXCL1	52	Tcctgcatcccccatagtta	Cttcaggaacagccaccagt
9	EAAT1	88	Ggccaagaagaaagtgcaga	Ggtcggagggtaaatccaag
10	EAAT2	58	Gcccaaatgaatggtgttgt	Gggtggctgtgaggctta
11	Kir4.1	39	Cagctctgctcctaactcctg	Aatacaccttggcaactgacg
12	AQP4	17	Gggaaattgggaaaaccatt	Gacatactcataaaggccaccag
