# Supplementary material for: Astrocyte senescence promotes glutamate toxicity in cortical neurons
Source: PLoS One. 2020 Jan 16;15(1):e0227887. doi: 10.1371/journal.pone.0227887 (PMC6964973; doi:10.1371/journal.pone.0227887)
Supplement: S2 Table — (PDF) [file pone.0227887.s007.pdf]

Sample	Raw Reads	Reads After Filtering and Trimming	Mapped Reads
**NS1**	23,809,882	23,403,727	21,062,807 (89.7%)
**NS2**	23,258,856	22,891,123	20,713,954 (90.1%)
**NS3**	20,585,129	20,228,224	18,234,258 (89.8%)
**NS4**	23,682,267	23,395,688	20,988,408 (89.3%)
**NS5**	24,312,598	23,918,805	21,615,591 (90.1%)
**NS6**	21,107,010	20,778,234	18,845,265 (90.4%)
**SEN1**	25,526,314	25,094,062	22,553,948 (89.5%)
**SEN2**	25,858,196	25,429,881	22,858,881(89.6%)
**SEN3**	23,236,065	22,894,407	20,594,937 (89.5%)
**SEN4**	23,682,267	23,331,471	21,023,811 (89.8%)
**SEN5**	25,643,811	25,238,390	22,749,668 (89.9%)
**SEN6**	23,633,802	23,248,316	20,898,565 (89.6%)
